# Verruca Vulgaris-Associated Cutaneous Horn: A Case Report

**DOI:** 10.7759/cureus.38962

**Published:** 2023-05-13

**Authors:** Elizabeth Edah, Dhruviben Patel, Jetvir Singh, Frederick Tiesenga

**Affiliations:** 1 Department of Surgery, All Saints University, Roseau, DMA; 2 Department of Surgery, St. George’s University, True Blue, GRD; 3 Department of Surgery, Windsor University School of Medicine, Cayon, KNA; 4 Department of General Surgery, West Suburban Medical Center, Chicago, USA

**Keywords:** actinic keratosis, seborrheic keratosis, squamous cell carcinoma (scc), verruca vulgaris, cutaneous horn

## Abstract

A cutaneous horn is a yellow or white-colored conical projection made up of complex keratin that arises from the surface of the skin. It is usually diagnosed clinically but requires histologic examination to rule out malignancy or determine the underlying lesion. Verruca vulgaris, a human papillomavirus-associated lesion, is a very common benign underlying lesion. We present a case of an 80-year-old female who presented with a cutaneous horn on a unique location, the proximal interphalangeal joint (PIP) of her left fourth digit. Post-excision biopsy revealed a diagnosis of a verruca vulgaris-associated cutaneous horn.

## Introduction

A cutaneous horn (cornu cutaneum) is a hyperkeratotic projection above the surface of the skin. They are so named as they resemble an animal’s horn physically but differ histologically as they consist of cornified proliferative keratinocytes without a bony component [1]. Cutaneous horns are more common in patients aged 60 to 70, especially those with fair skin. They can present as a single lesion or multiple lesions anywhere on the body but exhibit a predilection for sun-exposed areas like the head, ears, back of hands, and forearms [2]. Cutaneous horns can arise from benign, premalignant, or malignant skin lesions. The most common benign underlying lesion is seborrheic keratosis while the most common premalignant and malignant lesions are actinic keratoses and squamous cell carcinoma respectively [3]. Other common underlying lesions include but are not limited to molluscum contagiosum, hypertrophic lichen planus, and verruca vulgaris. Verruca vulgaris is a benign cutaneous wart produced by human papillomavirus (HPV) invasion of epithelial cells, with HPV-2, HPV-4, or HPV-40 being the most prevalent causative kinds. The lesions are usually located on the hands, fingers, knees, and elbows [4]. On rare occasions, verruca vulgaris can become keratinized and present as a cutaneous horn [5]. There is no other evidence of a verruca vulgaris-associated cutaneous horn on the proximal interphalangeal joint (PIP) in the literature, hence our case report focuses on the pathogenesis, current treatment modalities, and an occurrence in a novel place.

## Case presentation

**Figure 1:**
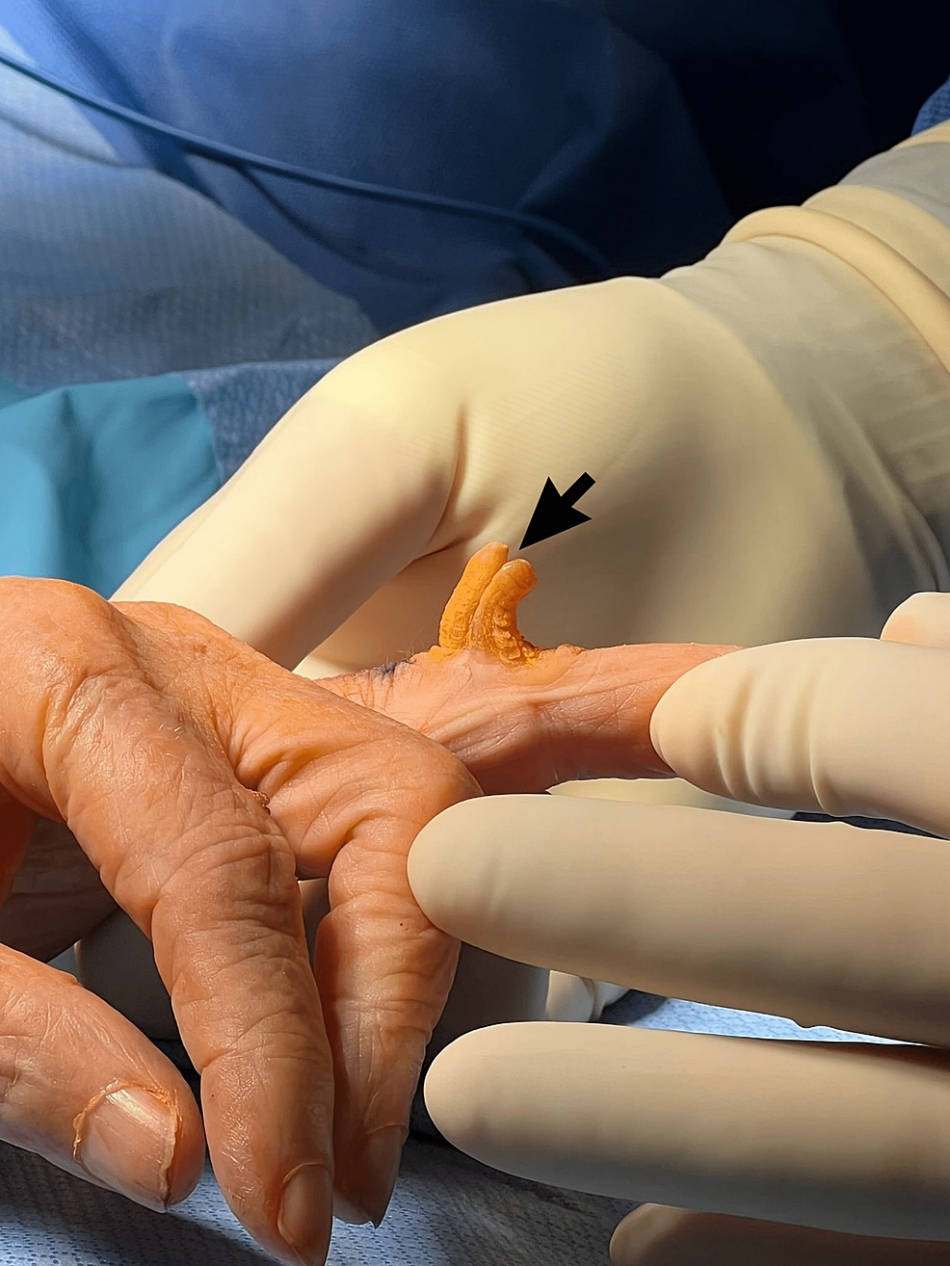
Image of the cutaneous lesion Image showing a double-horned lesion on the proximal interphalangeal joint of the left fourth finger of an 80-year-old female

**Figure 2:**
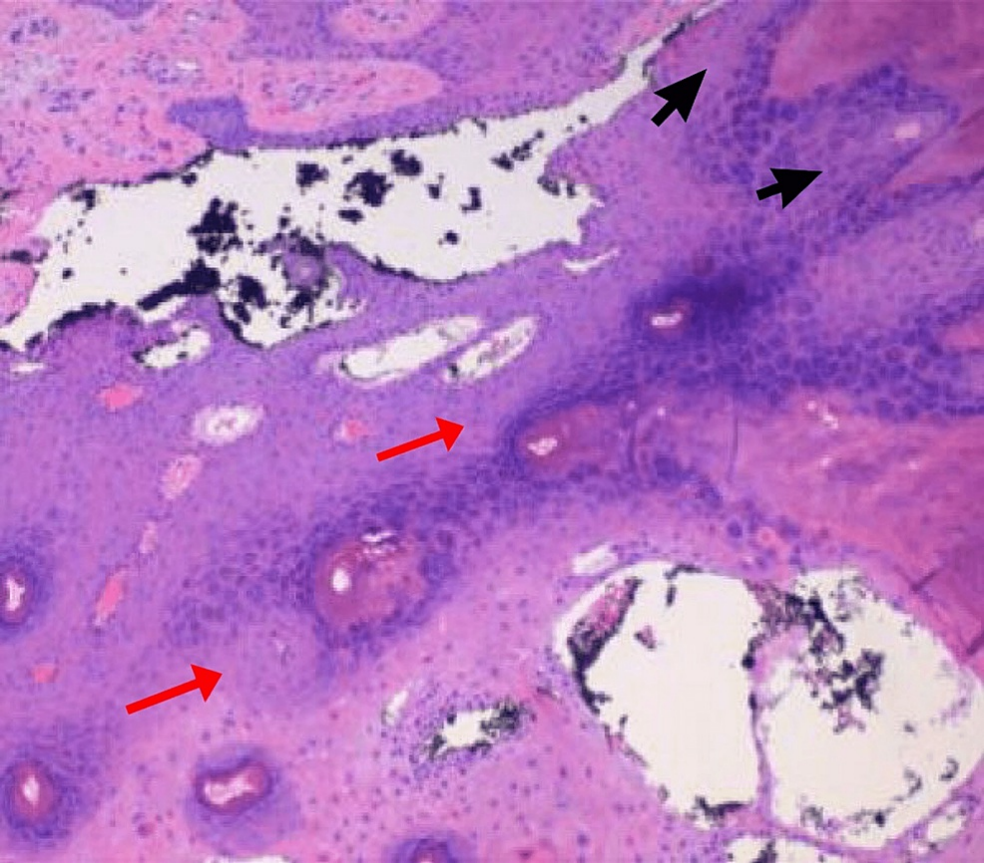
Histopathology image of the underlying lesion Tissue biopsy specimen of the underlying lesion showing thickening of the epidermis (red arrows) and papillary projections (black arrows) indicating papillomatosis of the epidermis.

An 80-year-old female with a past medical history of dementia, hypertension, and bipolar disorder was evaluated for a growth on her left fourth finger. The growth had been present for a year and she denied any family history of similar lesions. On examination, her vitals were within normal limits, and a double-horned, yellow-tan hard keratotic lesion was present on the dorsal surface of the PIP of her left fourth finger. The patient denied numbness, tingling, or any sensory abnormality on all fingers. The lesion was mobile and mildly tender with movement. She retained a full range of motion in her fingers and there was no enlargement of the cubital lymph nodes. The patient consented to surgery, the entire mass was excised at its base under local anesthesia with an oval incision 3 cm long and 2 cm wide down to the subcutaneous tissue of the left fourth finger (Figure 1). The patient was discharged on the same day and no complications were recorded after the scheduled follow-up a week after. The underlying lesion, which measured 1.9 x 1.5 x 1.0 cm, had epidermal thickening and papillomatosis on histology, which is typical of Verruca vulgaris (Figure 2).

## Discussion

Cornu cutaneum is an unusual hard conical projection made of compact keratin on the surface of the skin. They can arise from benign, premalignant, or malignant skin lesions [3]. They are more commonly found in the elderly population with an age range of 60 to 80 years and are more likely to be premalignant or malignant in these populations. The mean age of patients with premalignant or malignant base lesions was observed in a retrospective study to be 8.9 years greater than those with benign base lesions [3]. The cause of cutaneous horns remains unknown, but radiation exposure is implied to be a trigger as a greater percentage of cases occur on the face and hands or areas frequently exposed to sunlight [6].

Cutaneous horns usually arise due to an underlying epidermal lesion. The most common benign lesions are seborrheic keratosis, molluscum contagiosum, hypertrophic lichen planus, or verruca vulgaris. The most common premalignant lesions are actinic keratosis and keratoacanthoma, and squamous cell carcinoma is the most common malignant cause [7]. Most patients are usually asymptomatic like our patient but these lesions can sometimes be accompanied by pain. They typically do not differ in presentation clinically and biopsies serve as a tool to determine what type of underlying epidermal lesion exists under the horn [8].

According to the post-excision histopathology report, verruca vulgaris was confirmed as our patient’s underlying epidermal lesion. Verruca vulgaris, a human papillomavirus (HPV)-associated lesion, is commonly observed to present as a papule but can rarely present as a cutaneous horn by developing hyperkeratosis [1]. Cutaneous horns can be treated surgically or via laser ablation and management depends on the type of underlying lesion. For verruca vulgaris-associated cutaneous horns, complete excision and biopsy are the current standard of care, and most patients will require no additional therapy [3]. If the lesion extends beyond the margins of the biopsy specimen, modalities similar to that of verruca vulgaris that is not associated with a cutaneous horn with topical agents like salicylic acid, cantharidin, and cryotherapy are utilized [4,7]. For premalignant or malignant cases, wide local excision with appropriate margins is the preferred standard of care [9,10]. Cryosurgery, laser ablation, or electrocautery are additional alternative therapeutic options for small cutaneous horns and lesions with low malignant potential. They are particularly useful for cosmetic purposes because of the failure to preserve histologic specimens [8]. Cutaneous horns with a malignant lesion as the underlying lesion should also be evaluated for metastasis and adequate follow-up scheduled for the first three years after diagnosis [1,10].

## Conclusions

This case report adds to the current literature by describing an 80-year-old female presenting with a cutaneous horn on a unique location, the PIP of her left fourth digit, which was later diagnosed as a verruca vulgaris-associated lesion after a histologic examination. Cutaneous horns are usually diagnosed clinically but require a histologic examination to determine the underlying lesion and rule out malignancy. Cutaneous horns can arise from benign, premalignant, or malignant skin lesions. Management depends on the type of underlying lesion, with wide local excision with appropriate margins being the preferred standard of care for premalignant or malignant cases. For verruca vulgaris-associated cutaneous horns, biopsy after a complete excision can suffice, and most patients will require no additional therapy.
